# Meta-Analysis of 49 SNPs Covering 25,446 Cases and 41,106 Controls Identifies Polymorphisms in Hormone Regulation and DNA Repair Genes Associated with Increased Endometrial Cancer Risk

**DOI:** 10.3390/genes14030741

**Published:** 2023-03-17

**Authors:** Agneesh Pratim Das, Nisha Chaudhary, Shrishty Tyagi, Subhash M. Agarwal

**Affiliations:** 1 Bioinformatics Division, ICMR-National Institute of Cancer Prevention and Research, I-7, Sector-39, Noida 201301, India; 2 Multanimal Modi College, Chaudhary Charan Singh University, Modinagar 201204, India

**Keywords:** meta-analysis, single nucleotide polymorphism, endometrial cancer, odds ratio, DNA repair, hormone regulation

## Abstract

Endometrial cancer (EC) is among the most common gynecological disorders globally. As single nucleotide polymorphisms (SNPs) play an important role in the causation of EC, therefore, a comprehensive meta-analysis of 49 SNPs covering 25,446 cases and 41,106 controls was performed to identify SNPs significantly associated with increased EC risk. PubMed was searched to identify case control studies and meta-analysis was performed to compute the pooled odds ratio (OR) at 95% confidence interval (CI). Cochran’s Q-test and I^2^ were used to study heterogeneity, based on which either a random or a fixed effect model was implemented. The meta-analysis identified 11 SNPs (from 10 genes) to be significantly associated with increased EC risk. Among these, seven SNPs were significant in at least three of the five genetic models, as well as three of the polymorphisms (rs1801320, rs11224561, and rs2279744) corresponding to *RAD51*, *PGR*, and *MDM2* genes, which contained more than 1000 EC cases each and exhibited increased risk. The current meta-analysis indicates that polymorphisms associated with various hormone related genes—*SULT1A1* (rs1042028), *PGR* (rs11224561), and *CYP19A1* (rs10046 and rs4775936); DNA repair genes—*ERCC2* (rs1799793), *OGG1* (rs1052133), *MLH1* (rs1800734), and *RAD51* (rs1801320) as well as genes like *MDM2* (rs2279744), *CCND1* (rs9344), and *SERPINE1* (rs1799889), are significantly associated with increased EC risk.

## 1. Introduction

Endometrial cancer (EC) is a well-known cancer of the female reproductive system. It is the second most predominant and fourth leading reason of death from gynecological cancers amongst females globally [1]. Its development is dependent on both genetic and sporadic factors that may act as either causal agents or risk modifiers [2]. EC can be categorized into two types, i.e., I and II, which are based on the nature of the tumors (endometrioid and non-endometrioid). Among the two, type I is the most commonly found category (75–90%) [3]. Literature evidence suggests that the activation and/or inactivation of certain genes is important in the development of EC [4]. In this respect, the study of single nucleotide polymorphisms (SNPs) is important, as they contribute towards the aberrant activity of the genes by changing a single nucleotide within the gene sequence. Population-based case-control studies of candidate genes are routinely used for analyzing the genotypic distribution of SNPs in cancer patients and normal populations, which help in drawing conclusions about their role in cancer susceptibility. Moreover, since the outcomes of individual candidate gene-based population studies are often dissimilar in different populations, a meta-analysis allows us to pool the data from all such investigations and re-evaluate the hypothesis based on the previously known studies [5,6]. Although numerous studies exist that analyze the effect of individual polymorphisms on EC incidence, a comprehensive meta-analysis of a number of SNPs has not been reported yet. As meta-analysis is a powerful technique capable of generating statistically significant inferences from population-based evidence published in literature, therefore the PICO question was framed as, “Which single nucleotide polymorphisms confer increased risk in Endometrial cancer patients?” To conduct the above study, the PubMed database was queried to identify case-control studies of SNPs in EC. Thereafter, the allele, dominant, recessive, heterozygous, and homozygous models were used to pool the effect from these studies. The statistical model used for calculating the odds ratio (OR) was decided based on the heterogeneity across studies, and publication bias was evaluated by Begg’s funnel plot combined with Egger’s test. As a result, the current meta-analysis has identified 11 SNPs that are capable of increasing the risk of EC susceptibility and development. These high-risk SNPs were observed to be majorly associated with DNA repair and hormonal regulation.

## 2. Materials and Methods

**Figure 1:**
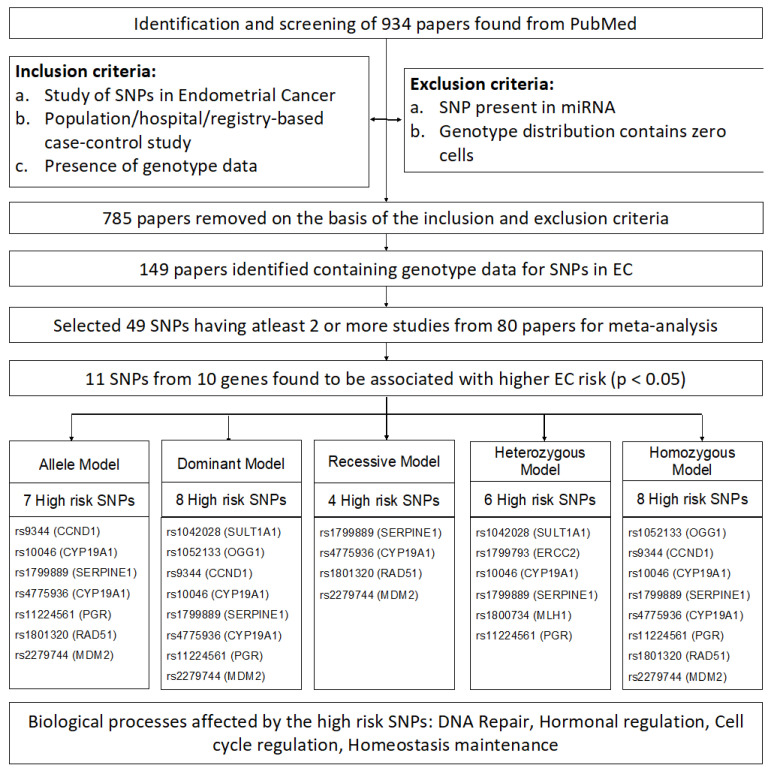
Workflow adopted in the current study.

The workflow adopted in the current work is given in Figure 1, as well as described in detail below.

### 2.1. Screening and Selection

The search phrases “SNP” or “single nucleotide polymorphism” and “endometrial cancer” or “endometrial carcinoma” were used to conduct a web-based text mining query of the PubMed database using the R package “RISMed” [7,8]. The search was limited until December 2021 and all the indexed papers were identified, screened, and selected by analyzing the results. The studies were chosen using the following inclusion and exclusion criteria: (a) the study must describe the association between SNPs and the risk of EC, (b) it is a population/hospital/registry-based case-control study, (c) it has publicly available genotype data to calculate OR with a 95% confidence interval (CI), and (d) papers containing SNPs in miRNA region or genotype distribution having zero cells were removed.

### 2.2. Data Collection

Two reviewers (NC and ST) retrieved data from the selected papers independently, which were then validated by APD. The data included the first author’s name, publication year, RSID, genomic location, gene, number of cases and controls, including genotype data, and genotyping techniques, as well.

### 2.3. Meta-Analysis

The risk association of the variant genotypes with EC was evaluated via five genetic models: allele, dominant, recessive, heterozygous, and homozygous, respectively. For each SNP having major and minor alleles ‘A’ and ‘a’ respectively, the models are defined as follows: allele model: a vs A, dominant model: aa + Aa vs AA, recessive model: aa vs Aa + AA, heterozygous model: Aa vs AA, and homozygous model: aa vs AA. The genotypic distributions of the SNPs as observed in the respective case-control studies were then used to calculate the five models. Thereafter, the relation between the SNPs and EC susceptibility was assessed using pooled OR with 95% CI. An SNP was identified as statistically significant with respect to its association with EC if the *p*-value of the pooled OR was less than 0.05. The I^2^ test for heterogeneity was used to identify the percentage of the total variance among the studies. A fixed effect (aka common effect) model was used to perform the meta-analysis if I^2^ was ≤ 50%, while in cases where I^2^ was > 50%, a random effects model was employed [9]. Funnel plots were used along with Egger’s test to measure publication bias. The ‘meta’, ‘dmetar’, and ‘tidyverse’ R packages were used for meta-analysis and generating the plots [10,11].

## 3. Results

**Figure 2:**
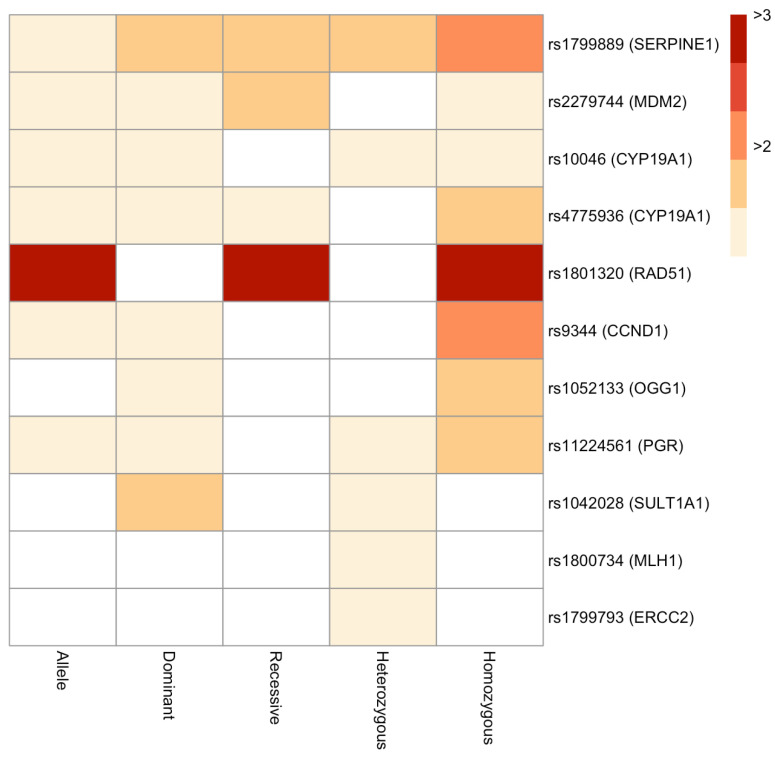
Heatmap representation of the 11 SNPs significantly associated with increased EC risk across all genetic models.

The PubMed search led to the identification of 934 papers, which were screened based on the inclusion and exclusion criteria, leading to the removal of 785 papers and the selection of 149 papers (Table S1). From these papers, only SNPs were selected for which at least two or more case-control studies were present. This led to the identification of 49 polymorphisms covering 25,446 cases and 41,106 controls from 80 studies (Table S2). Subsequently, a meta-analysis was undertaken to check the link between these 49 SNPs and EC risk. The pooled OR and 95% CI of each SNP were determined in all the models, and the heterogeneity across studies was also examined. It was observed that among the five genetic models, seven, eight, four, six, and eight SNPs exhibited significant association with increased EC risk in the allele, dominant, recessive, heterozygous, and homozygous models, respectively (Figure 1, Table 1, Table 2, Table 3, Table 4 and Table 5, Supplementary File S1). For ease of understanding, these SNPs and their corresponding genes have been depicted in the form of a heatmap of their pooled OR in each of the models (Figure 2). It was noted that the seven polymorphisms rs1799889 (*SERPINE1* aka Plasminogen activator inhibitor type 1 or *PAI1*), rs2279744 (*MDM2* aka murine double minute 2), rs10046 (*CYP19A1* aka cytochrome P450 family 19 sub-family A member 1), rs4775936 (*CYP19A1*), rs1801320 (*RAD51* aka RAD51 Recombinase), rs9344 (*CCND1* aka Cyclin D1), and rs11224561 (*PGR* aka progesterone receptor) were significantly associated with increased EC risk in at least 3 models. Additionally, the *RAD51* and *SERPINE1* gene polymorphisms (rs1801320 and rs1799889) exhibited the highest ORs across different genetic models. The results of the individual models have been described separately below.

In the allele model, seven SNPs (rs9344, rs10046, rs1799889, rs4775936, rs11224561, rs1801320, and rs2279744) were found to increase EC risk (Table 1, Supplementary File S1). The highest OR was observed in the case of the rs1801320 polymorphism of the *RAD51* gene (OR = 4.29, 95% CI = 2.02–9.11, *p* = 0.008642). In this model, two of the significant SNPs (rs1801320 and rs2279744) had a Q test *p*-value ≤ 0.01 and an I^2^ value >50% in the heterogeneity analysis. As a result, the random effects model was employed for the meta-analysis of these two SNPs, while for the rest of the five SNPs, the fixed effect model was used. 

In the dominant model, eight SNPs (rs1042028, rs1052133, rs9344, rs10046, rs1799889, rs4775936, rs11224561, and rs2279744) were identified as significant with increased pooled OR (Table 2, Supplementary File S1). In this model, the highest OR was observed in the case of the rs1799889 polymorphism of the *SERPINE1* gene (OR = 1.74, 95% CI = 1.23–2.47, *p* = 0.001878). All eight statistically significant SNPs had Q test *p*-value > 0.01 and I^2^ value less than 50%, and therefore pooled OR was computed using a fixed effect model. 

In the recessive model, only four SNPs (rs1799889, rs4775936, rs1801320, and rs2279744) were identified to increase risk significantly (Table 3, Supplementary File S1). Among these, rs1801320 and rs2279744 had Q test *p*-value < 0.01 and I^2^ > 50%. Therefore, the random effects model was used to pool the data for these two SNPs, while for the others, the fixed effect model was used. Similar to the allele model, rs1801320 exhibited the highest pooled OR value (OR = 10.03, 95% CI = 3.57–28.19, *p* = 0.005741). 

In the heterozygous model, six polymorphisms (rs1042028, rs1799793, rs10046, rs1799889, rs1800734, and rs11224561) were found to be associated with increased OR, all of which had Q values > 0.01 and I^2^ < 50% (Table 4, Supplementary File S1). Hence, for these SNPs, the pooled OR was computed using a fixed effect model. Similar to the dominant model, the highest OR (OR = 1.56, 95% CI = 1.08–2.25, *p* = 0.018038) was found for rs1799889.

In the homozygous model, eight SNPs (rs1052133, rs9344, rs10046, rs1799889, rs4775936, rs11224561, rs1801320, and rs2279744) exhibited a higher risk of EC (Table 5, Supplementary File S1). For seven of these polymorphisms, the pooled OR was computed using a fixed effect model, as they had Q values ≥ 0.01 and I^2^ < 50%. For the remaining SNP (rs1801320), the random effects model was used. Similar to the allele and recessive models, rs1801320 polymorphism of the *RAD51* gene exhibited the highest pooled OR value (OR = 7.44, 95% CI = 2.16–25.61, *p* = 0.014058).

## 4. Discussion

As genetic factors are known to be associated with EC susceptibility and progression, a systematic and thorough meta-analysis was performed in the current study. This led to the identification of polymorphisms in biological processes like hormonal regulation (*CYP19A1*, *PGR*, and *SULT1A1* i.e., sulfotransferase family 1A member 1) and DNA repair (*RAD51*, *OGG1* i.e., 8-oxoguanine DNA glycosylase, *MLH1* i.e., mutL homolog 1, and *ERCC2* i.e., ERCC excision repair 2), as well as proto-oncogene (*MDM2*), cell cycle regulator (*CCND1*), and the homeostasis-related gene (*SERPINE1*) to be linked with increased EC risk. These are discussed in detail below.

### 4.1. Hormone-Related Genes

EC is a hormone-related disease wherein genes *CYP19A1*, *PGR*, and *SULT1A1* have previously been linked with its development [12,13,14]. These genes are involved in various cellular processes like encoding the aromatase enzyme [15], transforming androgens to estrogens (estrogen biosynthesis) [16], inhibiting estrogen-induced abnormal growth, etc. Therefore, any change in the regular activity of these genes due to the advent of polymorphisms may prove to be detrimental to patient health [2,4,17]. The present meta-analysis has identified polymorphisms in these genes which are significantly associated with EC and are also supported with literature evidence. Additionally, two of the three genes described above, i.e., *CYP19A1* and *PGR*, have genome wide association study (GWAS)-based evidence that supports their role in EC susceptibility [18,19,20]. 

The *CYP19A1* gene is present in the steroid hormone biosynthesis pathway (KEGG hsa00140) and is primarily responsible for the production of the aromatase enzyme. This enzyme converts the androgen class of hormones into estrogen as a part of the estrogen biosynthesis and metabolism pathway [21]. Therefore, one of the most plausible avenues of EC risk estimation is the study of polymorphisms in genes involved in the biosynthesis and metabolism of steroid hormones [22]. For example, the proportion of endogenous estrogen, estradiol, may change due to the presence of functional variations in these genes, which may increase the risk of developing EC [23]. Two polymorphisms in the *CYP19A1* gene, rs10046 and rs4775936, have been analyzed in the current meta-analysis using 611 cases and 1373 controls. The rs10046 polymorphism corresponds to a C>T change at the 1558^th^ position, while rs4775936 is a G>A change upstream of the translational start site [23,24]. In the case of rs10046, for all the genetic models (except recessive), a substantial association between the polymorphism and EC risk was identified: allele (OR = 1.21, 95% CI = 1.06–1.39, *p* = 0.005794), dominant (OR = 1.37, 95% CI = 1.09–1.72, *p* = 0.007032) heterozygous (OR = 1.31, 95% CI = 1.03–1.67, *p* = 0.025399), and homozygous (OR = 1.5, 95% CI = 1.14–1.99, *p* = 0.004126) models (Table 1, Table 2, Table 3, Table 4 and Table 5). Additionally, Paynter et al., 2005 reported that rs10046 may have functional importance in the development of certain cancers (breast cancer) by influencing mRNA stability or translation termination control [23]. Similarly, the rs4775936 polymorphism was found to be significant in the allele (OR = 1.25, 95% CI = 1.09–1.44, *p* = 0.001213), dominant (OR = 1.3, 95% CI = 1.05–1.61, *p* = 0.015277), recessive (OR = 1.4, 95% CI = 1.11–1.77, *p* = 0.004165) and homozygous (OR = 1.6, 95% CI = 1.22–2.1, *p* = 0.000806) models (Table 1, Table 2, Table 3 and Table 5, Supplementary File S1). The occurrence of these SNPs is therefore critical, as they may result in alterations of the aromatase activity of *CYP19A1*, thereby leading to increased EC risk.

The *PGR* gene is a part of the estrogen signaling pathway (KEGG hsa04915) in humans, which interacts with the steroid hormone progesterone to prevent excessive estrogen stimulation and estrogen-induced proliferation [25]. Therefore, any alteration in the biological function of *PGR* due to polymorphisms may alter the progesterone-mediated tumor suppression, thereby increasing EC risk. The rs11224561 polymorphism of the PGR gene represents a C>T change in the 3′ flanking region, which was analyzed in the current study using 2425 cases and 2658 controls from the Shanghai Endometrial Cancer Study, China (SECS), Australian National Endometrial Cancer Study, Australia (ANECS), and the Leuven Endometrial Study, Belgium (LES). The most significant association for this polymorphism was in the homozygote genotype TT (OR = 1.55, 95% CI = 1.2–2.01, *p* = 0.000828; Table 5) in comparison to the heterozygote genotype CT (OR = 1.24, 95% CI = 1.07–1.45, *p* = 0.004969; Table 4) and the dominant model TT+TC (OR = 1.29, 95% CI = 1.11–1.49, *p* = 0.000710; Table 2) (Supplementary File S1). As the *PGR* gene is responsible for interacting with progesterone and maintaining hormonal regulation [26], the presence of this polymorphism may alter the gene function, thereby leading to a diseased state.

As mentioned in the above paragraphs, EC etiology and development is linked to both the expression and metabolism of estrogen. Sulfotransferase (SULT) catalyzes the sulphate conjugation of estrogen metabolites in order to excrete them through urine [27]. *SULT1A1* is one of the primary members of this SULT family that can metabolize estrone, estradiol, and their intermediate products like catechol estrogens [28].The 638G>A polymorphism (rs1042028) of the *SULT1A1* gene was also analyzed in the current study by pooling the genotype data from 312 cases and 345 controls. This SNP significantly increased EC risk in the heterozygous genotype AA (OR = 1.5, 95% CI = 1.06–2.14, *p* = 0.023277; Table 4) and the dominant model AA + GG (OR = 1.6, 95% CI = 1.17–2.21, *p* = 0.003737; Table 2) (Supplementary File S1). Literature evidence shows that the G to A change leads to an Arg213His replacement in the sulfotransferase gene, which reduces 85% enzyme activity [29]. Since this gene is involved in the transformation of procarcinogens, the presence of SNPs may lead to aberrant activity and carcinogenic developments [30].

### 4.2. DNA Repair-Related Genes

The current meta-analysis has identified polymorphisms in *RAD51*, *OGG1*, *MLH1*, and *ERCC2* genes to be significantly associated with increased EC risk. These genes are associated with the DNA double-stranded break repair process (DSB), base excision repair pathway (BER), DNA mismatch repair process (MMR), and nucleotide excision repair pathways (NER), respectively. As the functions of these genes involve DNA repair and maintenance of genome stability and integrity, any perturbation of these gene products may be lethal for various cellular processes [31].

The *RAD51* gene encodes an important protein of the homologous recombination repair process, which is involved in the repair of DNA lesions and DNA double-strand breaks [32,33]. In the present meta-analysis, the 135G > C polymorphism (rs1801320) of this gene was evaluated using 1130 cases and 1136 controls from four studies, where it is observed to be associated significantly (*p* < 0.05) associated with a high risk of EC in the allele (OR = 4.29, 95% CI = 2.02–9.11, *p* = 0.008642), recessive (OR = 10.03, 95% CI = 3.57–28.19, *p* = 0.005741), and homozygous (OR = 7.44, 95% CI = 2.16–25.61, *p* = 0.014058) models (Table 1, Table 3 and Table 5, Supplementary File S1). Due to its critical role in cellular maintenance, the presence of this polymorphism in the 5′ untranslated region of the gene may thus lead to an increased risk of DNA damage via decreased DNA damage repair in EC patients. 

The rs1052133 polymorphism in the *OGG1* gene corresponds to a C>G substitution at codon 326 that results in a serine-to-cysteine change. This SNP was analyzed in the present meta-analysis by pooling data from six studies having 1079 cases and 1323 controls. The meta-analysis shows that the homozygous genotype GG (OR = 1.65, 95% CI = 1.3–2.1, *p* = 0.000035; Table 5) and dominant model GG+GC vs CC (OR = 1.31, 95% CI = 1.09–1.56, *p* = 0.003153; Table 2) show significant association with an increased risk of EC (Supplementary File S1). Additionally, Aka et al., 2004 demonstrated that the Ser326Cys (CG) and Cys326Cys (GG) genotypes had slower DNA repair capabilities than the Ser326Ser (CC) genotype [34]. Since this gene is responsible for the repair of oxidatively generated DNA lesions (including single-strand breaks), the presence of polymorphisms may hinder this process and ultimately lead to an increased risk of EC. 

The −93G > A polymorphism (rs1800734) of the DNA mismatch repair (MMR) gene *MLH1* was analyzed in this study with respect to 754 cases and 864 controls from two studies. The heterozygous genotype GA was seen to be significantly related to enhanced EC risk (OR = 1.45, 95% CI = 1.19–1.81, *p* = 0.001132) (Table 4, Supplementary File S1). Since MMR is a key cellular process that keeps the genome stable by fixing mismatches during DNA replication, the polymorphism may lead to disruption in the MMR process and increase the frequency of cellular aberrations. The MMR process is deficient in around 30% of endometrial malignancies, wherein it is often caused by hypermethylation of the MLH1 promoter [35]. A total of ~3% of EC cases are also linked with lynch syndrome, which is caused by inherited mutations in the MMR genes [36].

Another DNA damage repair gene polymorphism that was also found significant in this meta-analysis is the Asp312Asn change in the *ERCC2* gene (rs1799793). This gene is a critical component of the basal transcription factor BTF2/TFIIH complex and is engaged in transcription-coupled NER [37]. A significant association between this polymorphism and EC risk was found in the heterozygote genotype GA (OR = 1.22, 95% CI = 1.02–1.45, *p* = 0.031413) by pooling the data from 1087 cases and 1141 controls, which suggests that the GA genotype contributes towards increased cancer risk (Table 4, Supplementary File S1). Within the TFIIH transcription factor, ERCC2 encodes a helicase that is evolutionarily conserved and dependent on ATP for its activity. This helicase is a part of the DNA unwinding process of the NER pathway and is involved in the identification and repair of DNA lesions containing large adducts and thymidine dimers [38,39]. Therefore, a polymorphism in this gene may disrupt helicase production and activity, thereby causing decreased DNA repair and increased chances of carcinogenesis.

### 4.3. Other Genes

The *SERPINE* gene is a known inhibitor of fibrinolysis, which acts via the suppression of tissue and urokinase plasminogen activators (tPA and uPA) [40]. In this study, the −816A>G polymorphism (rs1799889) was analyzed using two individual studies having 346 cases and 513 controls. Statistically significant association was observed in all five genetic models as given: allele (OR = 1.45, 95% CI = 1.19–1.77, *p* = 0.000244), dominant (OR = 1.74, 95% CI = 1.23–2.47, *p* = 0.001878), recessive (OR = 1.64, 95% CI = 1.19–2.27, *p* = 0.002664), heterozygous (OR = 1.56, 95% CI = 1.08–2.25, *p* = 0.018038), and homozygous (OR = 2.23, 95% CI = 1.46–3.42, *p* = 0.000231) models (Table 1, Table 2, Table 3, Table 4 and Table 5, Supplementary File S1). The variant homozygote genotype GG exhibited the highest overall cancer risk when compared to the other models, indicating a significant association of the variant allele with EC. The occurrence of this polymorphism in the gene’s promoter region may increase the plasminogen activator system’s pericellular activity [41,42], which is required for cancer cells to migrate and thereby increases the risk for cancer development. 

The *MDM2* T309G polymorphism (rs2279744) is widely studied in gynecological cancers like cervical, ovarian, and endometrial cancer [43]. With respect to the current meta-analysis, the effect of *MDM2* 309 was analyzed in 2233 cases and 7164 controls from 10 case-control studies, including global registries like the Nurses’ Health Study (NHS) and the Molecular Markers in Treatment of Endometrial Cancer (MoMaTEC). This SNP was observed to be significantly associated with increased EC risk in the allele (OR = 1.26, 95% CI = 1.05–1.52, Supplementary File S1), dominant (OR = 1.15, 95% CI = 1.04–1.28, *p* = 0.007404), recessive (OR = 1.64, 95% CI = 1.18–2.26, *p* = 0.007311), and homozygous (OR = 1.43, 95% CI = 1.23–1.66, *p* = 0.000004) models (Table 1, Table 2, Table 3 and Table 5, Supplementary File S1). MDM2 is an E3 ubiquitin ligase that inhibits the function of the p53 tumor suppressor protein, both by ubiquitination and direct protein binding [44]. Subsequently, the lowering of p53 levels may lead to the development of a carcinogenic impact [45]. The presence of SNP309 in the promoter region of *MDM2* may thus have a functional influence on the elevation of MDM2 protein levels, thereby affecting p53 tumor suppressor efficacy [46]. 

CCND1 protein regulates proliferation, differentiation, and transcriptional control via its role in the G1 to S phase transition in the cell cycle [47]. Excessive cellular proliferation resulting from CCND1 overexpression is a hallmark of a variety of malignancies, including EC [48,49]. The 870 G>A polymorphism (rs9344) in cyclin D1 was studied by pooling the data from two studies having 268 cases and 444 controls. The A allele of this polymorphism (OR = 1.4, 95% CI = 1.13–1.74, *p* = 0.002280) exhibits a significant relationship with increased EC risk, along with the dominant model (OR = 1.46, 95% CI = 1.02–2.07, *p* = 0.036328) and homozygous AA genotype (OR = 1.98, 95% CI = 1.28–3.06, *p* = 0.002235) (Table 1, Table 2 and Table 5, Supplementary File S1). Given the necessity of cell cycle regulation for maintaining genomic integrity [49], polymorphisms in this gene may regulate processes that affect DNA repair effectiveness, thereby resulting in disease onset. 

Overall, this comprehensive meta-analysis has covered several SNPs in EC. Additionally, the use of five genetic models allowed the existing data to be analyzed from different perspectives regarding the effect of the variant allele on disease susceptibility. Additionally, all risk estimates calculated in the current study were pooled, and publication bias was also not observed, which provides confidence that the results are meaningful.

### 4.4. Limitations

However, this meta-analysis does have some limitations, one of which is that the literature search was performed only on MEDLINE through PubMed. Additionally, few of the SNPs reported to confer increased cervical cancer susceptibility in this study have been obtained by pooling the data from only two studies. Therefore, for these SNPs, more case-control-based studies need to be performed, either in other populations or countries so that a more robust inference can be drawn regarding the association of these SNPs with EC. Additionally, in comparison to candidate gene association studies, which are performed on a limited number of pre-selected genes/pathways of interest, increased attention is being given to GWAS as they investigate genetic variations throughout the whole genome. Therefore, they are able to address some of the limitations of candidate gene studies, such as the insufficient coverage of variants in the selected genes as well as the identification of variants in unknown pathways [50]. GWAS also provides additional observations and inferences like identifying a genetic correlation between traits and determining confounding factors for disease development [51]. In the case of EC, a number of GWAS have been performed in the past that have led to the identification of new susceptibility loci and increased EC risk regions in the genome [18]. However, they have been performed mostly in European populations, and thus, candidate gene studies in different global populations may provide additional insights. 

## 5. Conclusions

The current meta-analysis found evidence of the association between 11 SNPs (from 10 genes) and increased EC risk. It is already known that EC is a hormone-related disorder. This meta-analysis has further demonstrated that along with polymorphisms in estrogen and progesterone hormone-related genes like *SULT1A1*, *PGR*, and *CYP19A1*, the SNPs in DNA damage repair genes like *ERCC2*, *OGG1*, *MLH1*, and *RAD51* are also significantly associated with increased EC risk. Apart from these SNPs, cellular growth and proliferation-related genetic polymorphisms like *CCND1* and *MDM2* were also found to be associated with higher EC risk. The current study has thus highlighted a set of polymorphisms from a wide variety of cellular and molecular processes that are important concerning EC and should be further studied globally to ascertain their effect on different populations and ethnic groups worldwide.

## Figures and Tables

**Table 1:** Meta-analysis results of the SNPs significantly associated with increased EC risk in the allele model.

RSID	Model	Study	OR (95% CI)	Weight (%)	Cochran’s Q	I^2^	T^2^	Pooled OR (95% CI)	*p*-Value	Egger’s *p*-Value
rs9344	Fixed	Kang et al. (2005)	1.69 (1.14–2.5)	27.83	0.26	21.45	0.01	1.4 (1.13–1.74)	0.002280	NA
		Ashton et al. (2008)	1.29 (0.99–1.67)	72.17						
rs10046	Fixed	Paynter et al. (2005)	1.22 (0.98–1.51)	39.43	0.94	0	0	1.21 (1.06–1.39)	0.005794	NA
		Lundin et al. (2012)	1.21 (1.01–1.44)	60.57						
rs1799889	Fixed	Gilabert-Estellés et al. (2012)	1.44 (1.1–1.88)	53.98	0.91	0	0	1.45 (1.19–1.77)	0.000244	NA
		Su et al. (2011)	1.47 (1.1–1.97)	46.02						
rs4775936	Fixed	Paynter et al. (2005)	1.28 (1.03–1.59)	39.47	0.79	0	0	1.25 (1.09–1.44)	0.001213	NA
		Lundin et al. (2012)	1.23 (1.03–1.47)	60.53						
rs11224561	Fixed	Xu_SECS et al. (2009)	1.15 (1–1.31)	55.99	0.71	0	0	1.19 (1.08–1.31)	0.000572	0.74
		O’Mara_ANECS et al. (2011)	1.26 (1.06–1.49)	33.81						
		O’Mara_LES et al. (2011)	1.2 (0.89–1.63)	10.2						
rs1801320	Random	Krupa et al. (2011)	7.23 (3.2–16.35)	15.69	0	90.72	0.18	4.29 (2.02–9.11)	0.008642	0.24
		Michalska et al. (2014)	2.55 (2.16–3)	29.58						
		Romanowicz-Makowska et al. (2012)	3.81 (2.87–5.05)	27.54						
		Smolarz et al. (2011)	6.32 (4.68–8.53)	27.19						
rs2279744	Random	Walsh et al. (2007)	1.38 (0.87–2.19)	7.2	0.01	58.26	0.05	1.26 (1.05–1.52)	0.019979	0.29
		Terry_NHS et al. (2008)	1.21 (1.04–1.41)	13.47						
		Ashton et al. (2009)	1.13 (0.86–1.48)	10.98						
		Ueda et al. (2009)	1.2 (0.83–1.74)	8.86						
		Nunobiki et al. (2009)	1.17 (0.79–1.73)	8.35						
		Knappskog_Haukeland et al. (2012)	1.06 (0.91–1.23)	13.47						
		Knappskog_MoMaTEC et al. (2012)	1.18 (1.02–1.36)	13.59						
		Zajac et al. (2012)	2.67 (1.83–3.9)	8.69						
		Yoneda et al. (2013)	1.33 (0.97–1.83)	9.94						
		Okamoto et al. (2015)	0.96 (0.53–1.72)	5.46						

SECS: Shanghai Endometrial Cancer Study; ANECS: Australian National Endometrial Cancer Study; LES: Leuven Endometrial Study; NHS: Nurses’ Health Study; MoMaTEC: Molecular Markers in Treatment of Endometrial Cancer.

**Table 2:** Meta-analysis results of the SNPs significantly associated with increased EC risk in the dominant model.

RSID	Model	Study	OR (95% CI)	Weight (%)	Cochran’s Q	I^2^	T^2^	Pooled OR (95% CI)	*p*-Value	Egger’s *p*-Value
rs1042028	Fixed	Gulyaeva et al. (2008)	1.28 (0.81–2.02)	55.09	0.17	47.01	0.05	1.6 (1.17–2.21)	0.003737	NA
		Hirata et al. (2008)	2 (1.28–3.14)	44.91						
rs1052133	Fixed	Krupa et al. (2011)	0.84 (0.26–2.7)	2.87	0.34	11.7	0.05	1.31 (1.09–1.56)	0.003153	0.64
		Cincin et al. (2012)	1.95 (1.16–3.26)	9.56						
		Smolarz et al. (2018)	1.33 (1.04–1.71)	50.79						
		Sobczuk et al. (2012)	1.26 (0.69–2.28)	8.92						
		Romanowicz-Makowska et al. (2011)	1.39 (0.86–2.25)	13.19						
		Hosono et al. (2013)	0.85 (0.51–1.42)	14.67						
rs9344	Fixed	Kang et al. (2005)	1.73 (0.86–3.47)	24.22	0.57	0	0	1.46 (1.02–2.07)	0.036328	NA
		Ashton et al. (2008)	1.37 (0.91–2.06)	75.78						
rs10046	Fixed	Paynter et al. (2005)	1.58 (1.08–2.33)	33.22	0.35	0	0.01	1.37 (1.09–1.72)	0.007032	NA
		Lundin et al. (2012)	1.26 (0.95–1.67)	66.78						
rs1799889	Fixed	Gilabert-Estellés et al. (2012)	1.75 (1.12–2.73)	60.17	0.98	0	0	1.74 (1.23–2.47)	0.001878	NA
		Su et al. (2011)	1.73 (0.98–3.06)	39.83						
rs4775936	Fixed	Paynter et al. (2005)	1.53 (1.07–2.18)	34.08	0.26	21.76	0.01	1.3 (1.05–1.61)	0.015277	NA
		Lundin et al. (2012)	1.18 (0.91–1.55)	65.92						
rs11224561	Fixed	Xu_SECS et al. (2009)	1.45 (1.08–1.95)	22.72	0.57	0	0	1.29 (1.11–1.49)	0.000710	0.96
		O’Mara_ANECS et al. (2011)	1.27 (1.05–1.53)	58.98						
		O’Mara_LES et al. (2011)	1.14 (0.8–1.61)	18.31						
rs2279744	Fixed	Walsh et al. (2007)	1.09 (0.57–2.1)	2.57	0.71	0	0.03	1.15 (1.04–1.28)	0.007404	0.41
		Terry_NHS et al. (2008)	1.15 (0.94–1.41)	25.2						
		Ashton et al. (2009)	1.14 (0.79–1.65)	7.83						
		Ueda et al. (2009)	0.81 (0.42–1.56)	2.99						
		Nunobiki et al. (2009)	0.71 (0.35–1.42)	2.82						
		Knappskog_Haukeland et al. (2012)	1.1 (0.9–1.36)	25.64						
		Knappskog_MoMaTEC et al. (2012)	1.24 (1.01–1.51)	25.49						
		Zajac et al. (2012)	1.68 (0.89–3.17)	2.15						
		Yoneda et al. (2013)	1.42 (0.86–2.36)	3.78						
		Okamoto et al. (2015)	0.81 (0.32–2.01)	1.52						

**Table 3:** Meta-analysis results of the SNPs significantly associated with increased EC risk in the recessive model.

RSID	Model	Study	OR (95% CI)	Weight (%)	Cochran’s Q	I^2^	T^2^	Pooled OR (95% CI)	*p*-Value	Egger’s *p*-Value
rs1799889	Fixed	Gilabert-Estellés et al. (2012)	1.59 (0.99–2.58)	47.02	0.87	0	0	1.64 (1.19–2.27)	0.002664	NA
		Su et al. (2011)	1.68 (1.09–2.61)	52.98						
rs4775936	Fixed	Paynter et al. (2005)	1.25 (0.88–1.77)	46.43	0.38	0	0.01	1.4 (1.11–1.77)	0.004165	NA
		Lundin et al. (2012)	1.54 (1.13–2.1)	53.57						
rs1801320	Random	Krupa et al. (2011)	16 (3.22–79.56)	10.63	0	90.68	0.31	10.03 (3.57–28.19)	0.005741	0.33
		Michalska et al. (2014)	4.68 (3.67–5.97)	31.94						
		Romanowicz-Makowska et al. (2012)	10.77 (6.98–16.62)	28.98						
		Smolarz et al. (2011)	18.45 (11.63–29.29)	28.45						
rs2279744	Random	Walsh et al. (2007)	2.55 (1.06–6.1)	6.45	0	67.82	0.14	1.64 (1.18–2.26)	0.007311	0.18
		Terry_NHS et al. (2008)	1.57 (1.17–2.1)	13.3						
		Ashton et al. (2009)	1.23 (0.73–2.08)	10.25						
		Ueda et al. (2009)	1.91 (1.04–3.49)	9.24						
		Nunobiki et al. (2009)	2 (1.04–3.83)	8.71						
		Knappskog_Haukeland et al. (2012)	1.01 (0.74–1.38)	13.07						
		Knappskog_MoMaTEC et al. (2012)	1.24 (0.93–1.64)	13.4						
		Zajac et al. (2012)	4.67 (2.7–8.08)	9.94						
		Yoneda et al. (2013)	1.49 (0.88–2.53)	10.25						
		Okamoto et al. (2015)	1.14 (0.41–3.15)	5.38						

**Table 4:** Meta-analysis results of the SNPs significantly associated with increased EC risk in the heterozygous model.

RSID	Model	Study	OR (95% CI)	Weight (%)	Cochran’s Q	I^2^	T^2^	Pooled OR (95% CI)	*p*-Value	Egger’s *p*-Value
rs1042028	Fixed	Gulyaeva et al. (2008)	1.29 (0.77–2.17)	50.16	0.43	0	0.01	1.5 (1.06–2.14)	0.023277	NA
		Hirata et al. (2008)	1.72 (1.06–2.78)	49.84						
rs1799793	Fixed	Weiss_CARE et al. (2005)	1.2 (0.89–1.62)	35.23	0.93	0	0	1.22 (1.02–1.45)	0.031413	NA
		Doherty_SEER et al. (2011)	1.22 (0.98–1.52)	64.77						
rs10046	Fixed	Paynter et al. (2005)	1.6 (1.07–2.41)	32.24	0.23	31.39	0.02	1.31 (1.03–1.67)	0.025399	NA
		Lundin et al. (2012)	1.18 (0.87–1.58)	67.76						
rs1799889	Fixed	Gilabert-Estellés et al. (2012)	1.61 (1.01–2.56)	60.24	0.83	0	0	1.56 (1.08–2.25)	0.018038	NA
		Su et al. (2011)	1.48 (0.82–2.68)	39.76						
rs1800734	Fixed	Beiner et al. (2006)	1.51 (1.2–1.91)	91	0.18	45.51	0.09	1.45 (1.16–1.81)	0.001132	NA
		Poplawski et al. (2015)	0.81 (0.34–1.94)	9						
rs11224561	Fixed	Xu_SECS et al. (2009)	1.42 (1.04–1.94)	22.11	0.47	0	0.01	1.24 (1.07–1.45)	0.004969	0.88
		O’Mara_ANECS et al. (2011)	1.24 (1.02–1.51)	59.13						
		O’Mara_LES et al. (2011)	1.05 (0.73–1.51)	18.76						

CARE: Women’s Contraceptive and Reproductive Experiences Study; SEER: Surveillance, Epidemiology and End Results Program.

**Table 5:** Meta-analysis results of the SNPs significantly associated with increased EC risk in the homozygous model.

RSID	Model	Study	OR (95% CI)	Weight (%)	Cochran’s Q	I^2^	T^2^	Pooled OR (95% CI)	*p*-Value	Egger’s *p*-Value
rs1052133	Fixed	Krupa et al. (2011)	0.96 (0.06–16.25)	0.93	0.1	45.58	0.17	1.65 (1.3–2.1)	0.000035	0.45
		Cincin et al. (2012)	0.65 (0.13–3.32)	3.70						
		Smolarz et al. (2018)	1.93 (1.46–2.55)	67.50						
		Sobczuk et al. (2012)	3.03 (0.75–12.16)	2.33						
		Romanowicz-Makowska et al. (2011)	1.86 (0.65–5.32)	4.99						
		Hosono et al. (2013)	0.75 (0.39–1.42)	20.55						
rs9344	Fixed	Kang et al. (2005)	3.08 (1.34–7.05)	22.64	0.21	35.07	0.08	1.98 (1.28–3.06)	0.002235	NA
		Ashton et al. (2008)	1.66 (0.99–2.78)	77.36						
rs10046	Fixed	Paynter et al. (2005)	1.55 (0.99–2.43)	38.47	0.87	0	0	1.5 (1.14–1.99)	0.004126	NA
		Lundin et al. (2012)	1.48 (1.03–2.11)	61.53						
rs1799889	Fixed	Gilabert-Estellés et al. (2012)	2.2 (1.24–3.92)	53.79	0.95	0	0	2.23 (1.46–3.42)	0.000231	NA
		Su et al. (2011)	2.26 (1.2–4.26)	46.21						
rs4775936	Fixed	Paynter et al. (2005)	1.61 (1.05–2.49)	40.12	0.95	0	0	1.6 (1.22– 2.1)	0.000806	NA
		Lundin et al. (2012)	1.59 (1.11–2.26)	59.88						
rs11224561	Fixed	Xu_SECS et al. (2009)	1.48 (1.08–2.01)	70.92	0.63	0	0.02	1.55 (1.2–2.01)	0.000828	0.2
		O’Mara_ANECS et al. (2011)	1.59 (0.93–2.7)	23.77						
		O’Mara_LES et al. (2011)	2.38 (0.94–6.04)	5.31						
rs1801320	Random	Krupa et al. (2011)	25.33 (4.48–143.32)	12.49	0	83.01	0.48	7.44 (2.16–25.61)	0.014058	0.19
		Michalska et al. (2014)	3.72 (2.77–5)	31.26						
		Romanowicz-Makowska et al. (2012)	5.41 (3.22–9.09)	28.57						
		Smolarz et al. (2011)	13 (7.27–23.24)	27.69						
rs2279744	Fixed	Walsh et al. (2007)	2.29 (0.89–5.89)	2.15	0.23	23.41	0.07	1.43 (1.23–1.66)	0.000004	0.41
		Terry_NHS et al. (2008)	1.6 (1.17–2.18)	22.32						
		Ashton et al. (2009)	1.29 (0.73–2.26)	7.87						
		Ueda et al. (2009)	1.36 (0.62–2.98)	3.97						
		Nunobiki et al. (2009)	1.27 (0.55–2.93)	3.6						
		Knappskog_Haukeland et al. (2012)	1.07 (0.77–1.49)	24.95						
		Knappskog_MoMaTEC et al. (2012)	1.36 (1–1.85)	24.84						
		Zajac et al. (2012)	3.5 (1.73–7.08)	2.87						
		Yoneda et al. (2013)	1.76 (0.93–3.3)	5.36						
		Okamoto et al. (2015)	0.95 (0.29–3.12)	2.08						

## Data Availability

Provided in the manuscript.

## Supplementary Materials

The following supporting information can be downloaded at: https://www.mdpi.com/article/10.3390/genes14030741/s1, Table S1: Papers not selected along with their reasons; Table S2: Characteristics of the studies included in the meta-analysis; Supplementary File S1: Forest plots of 11 significantly high-risk SNPs studied under different genetic models.
