# Supplementary material for: Meta-Analysis of 49 SNPs Covering 25,446 Cases and 41,106 Controls Identifies Polymorphisms in Hormone Regulation and DNA Repair Genes Associated with Increased Endometrial Cancer Risk
Source: Genes (Basel). 2023 Mar 17;14(3):741. doi: 10.3390/genes14030741 (PMC10048726; doi:10.3390/genes14030741)

Supplementary File 1: Forest plots of 11 significantly high-risk SNPs studied under different genetic models

a) rs1799889 (SERPINE1)

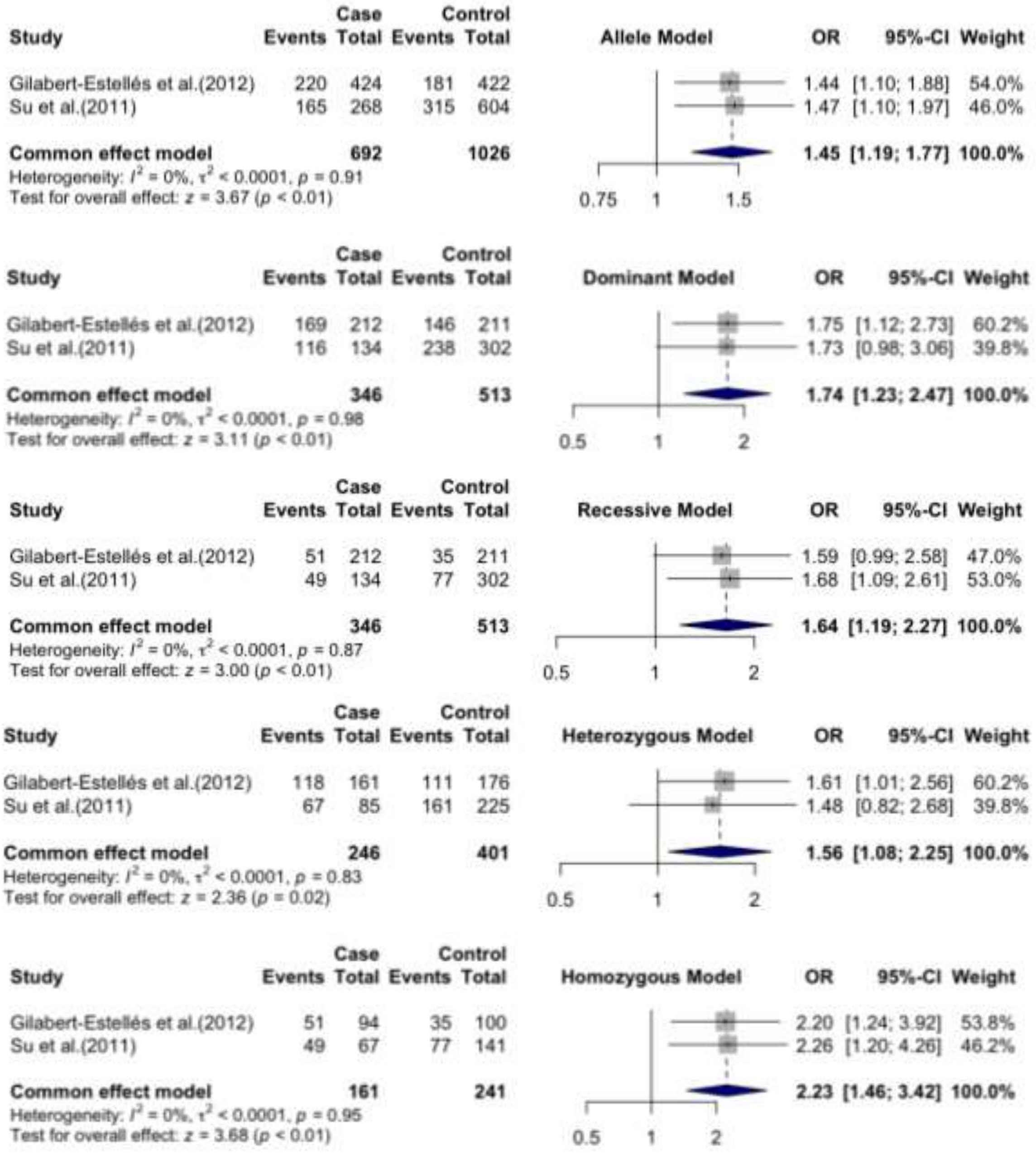

b) rs2279744 (MDM2)

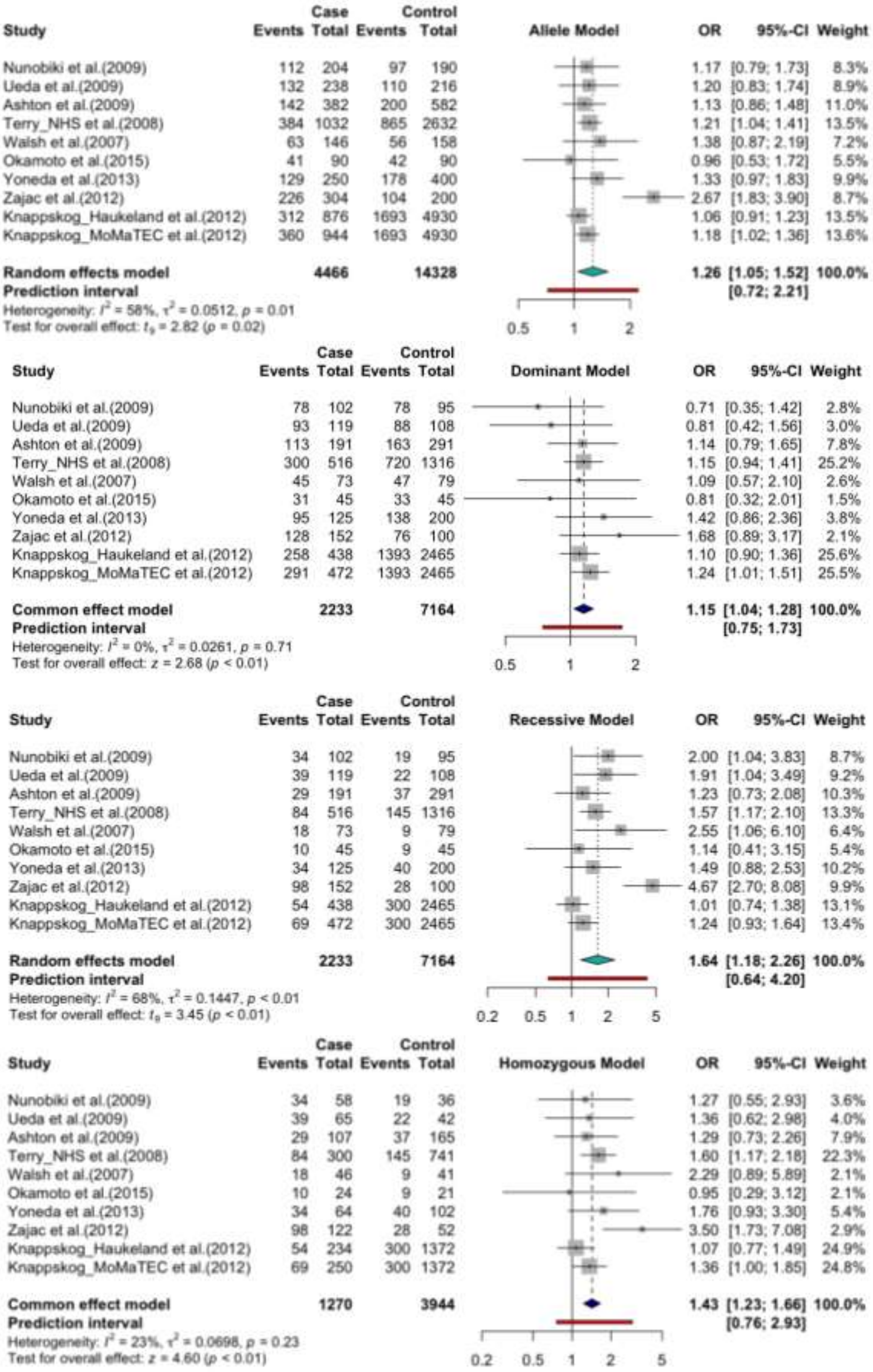

c) rs10046 (CYP19A1)

Study	Case Events	Case Total	Control Events	Control Total	OR	95%-CI	Weight
Paynter_NHS et al.(2005)	245	440	674	1328	1.22	[0.98; 1.51]	39.4%
Lundin_NHSDS+WHS et al.(2012)	390	782	641	1418	1.21	[1.01; 1.44]	60.6%
**Common effect model**		**1222**		**2746**	**1.21**	**[1.06; 1.39]**	**100.0%**

Heterogeneity: $I^2 = 0\%$, $\tau^2 < 0.0001$, $p = 0.94$
Test for overall effect: $z = 2.76$ ($p < 0.01$)

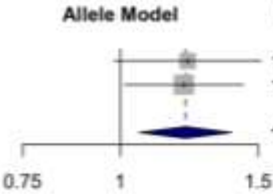

Study	Case Events	Case Total	Control Events	Control Total	OR	95%-CI	Weight
Paynter_NHS et al.(2005)	181	220	495	664	1.58	[1.08; 2.33]	33.2%
Lundin_NHSDS+WHS et al.(2012)	295	391	503	709	1.26	[0.95; 1.67]	66.8%
**Common effect model**		**611**		**1373**	**1.37**	**[1.09; 1.72]**	**100.0%**

Heterogeneity: $I^2 = 0\%$, $\tau^2 = 0.0082$, $p = 0.35$
Test for overall effect: $z = 2.70$ ($p < 0.01$)

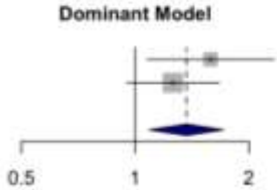

Study	Case Events	Case Total	Control Events	Control Total	OR	95%-CI	Weight
Paynter_NHS et al.(2005)	117	156	316	485	1.60	[1.07; 2.41]	32.2%
Lundin_NHSDS+WHS et al.(2012)	200	296	365	571	1.18	[0.87; 1.58]	67.8%
**Common effect model**		**452**		**1056**	**1.31**	**[1.03; 1.67]**	**100.0%**

Heterogeneity: $I^2 = 31\%$, $\tau^2 = 0.0204$, $p = 0.23$
Test for overall effect: $z = 2.24$ ($p = 0.03$)

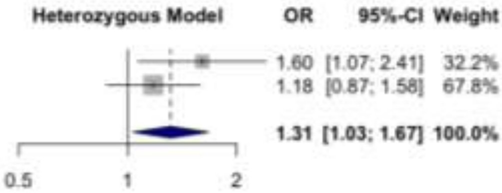

Study	Case Events	Case Total	Control Events	Control Total	OR	95%-CI	Weight
Paynter_NHS et al.(2005)	64	103	179	348	1.55	[0.99; 2.43]	38.5%
Lundin_NHSDS+WHS et al.(2012)	95	191	138	344	1.48	[1.03; 2.11]	61.5%
**Common effect model**		**294**		**692**	**1.50**	**[1.14; 1.99]**	**100.0%**

Heterogeneity: $I^2 = 0\%$, $\tau^2 < 0.0001$, $p = 0.87$
Test for overall effect: $z = 2.87$ ($p < 0.01$)

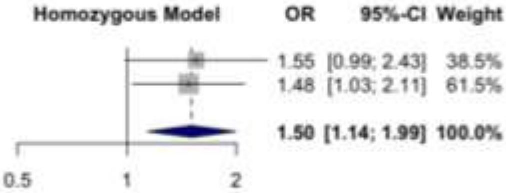

d) rs4775936 (CYP19A1)

Study	Case Events	Case Total	Control Events	Control Total	OR	95%-CI	Weight
Paynter_NHS et al.(2005)	228	440	606	1328	1.28	[1.03; 1.59]	39.5%
Lundin_NHSDS+WHS et al.(2012)	363	782	585	1418	1.23	[1.03; 1.47]	60.5%
**Common effect model**		**1222**		**2746**	**1.25**	**[1.09; 1.44]**	**100.0%**

Heterogeneity: $I^2 = 0\%$, $\tau^2 < 0.0001$, $p = 0.79$
Test for overall effect: $z = 3.24$ ($p < 0.01$)

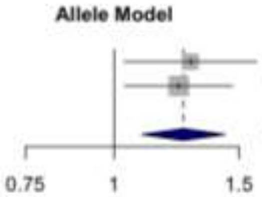

Study	Case Events	Case Total	Control Events	Control Total	OR	95%-CI	Weight
Paynter_NHS et al.(2005)	170	220	458	664	1.53	[1.07; 2.18]	34.1%
Lundin_NHSDS+WHS et al.(2012)	274	391	471	709	1.18	[0.91; 1.55]	65.9%
**Common effect model**		**611**		**1373**	**1.30**	**[1.05; 1.61]**	**100.0%**

Heterogeneity: $I^2 = 22\%$, $\tau^2 = 0.0128$, $p = 0.26$
Test for overall effect: $z = 2.43$ ($p = 0.02$)

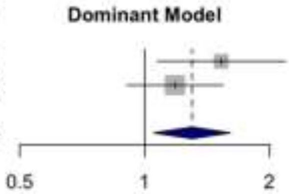

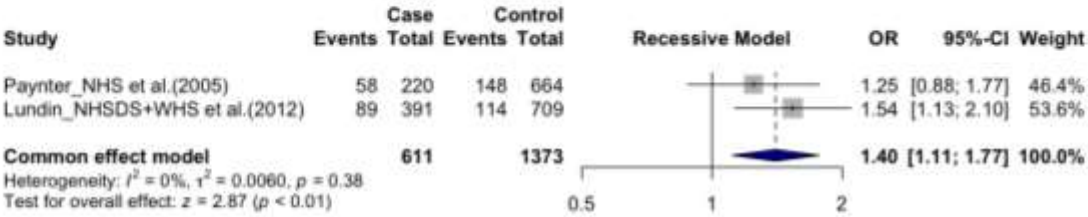

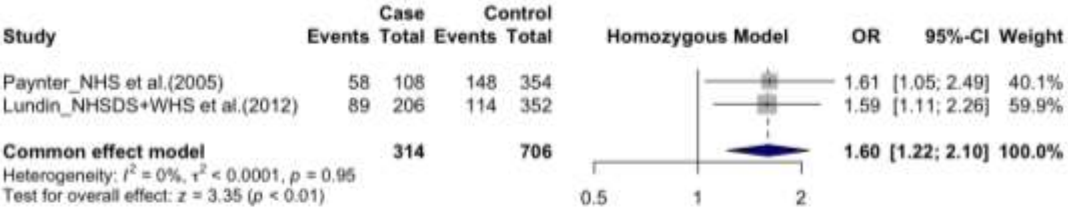

e) rs1801320 (RAD51)

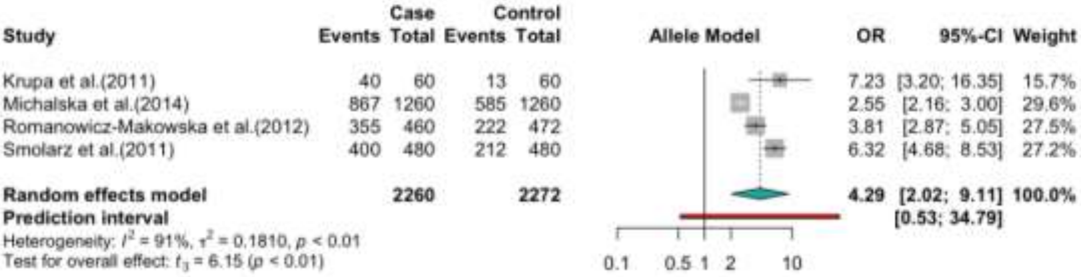

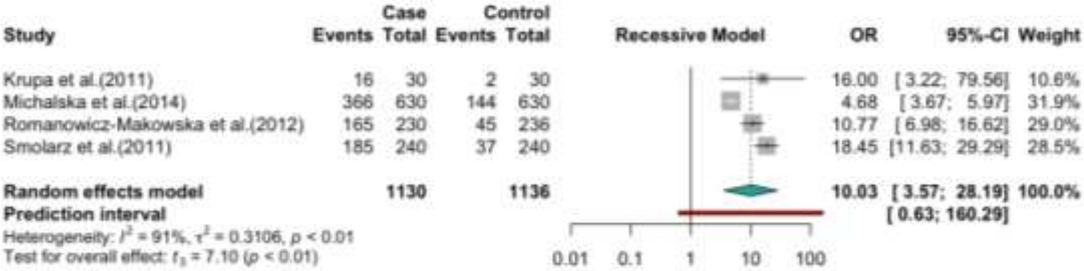

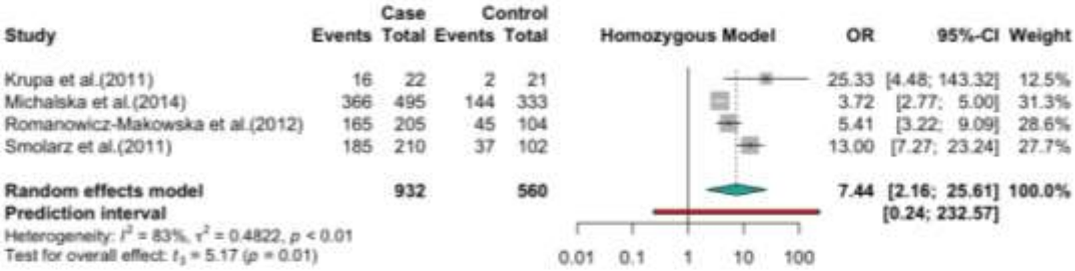

f) rs9344 (CCND1)

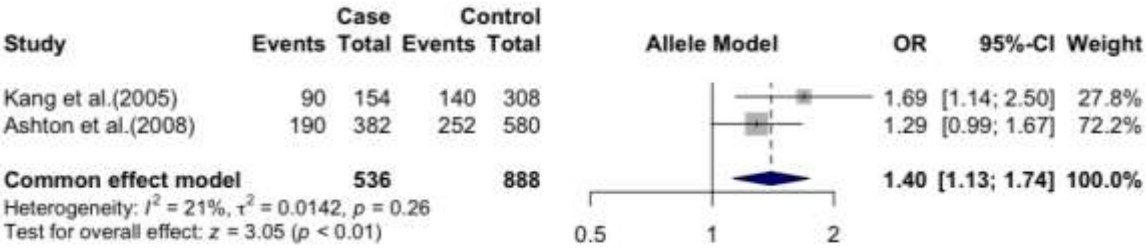

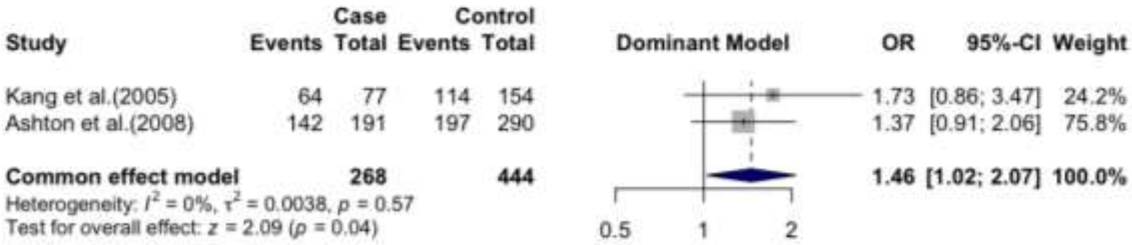

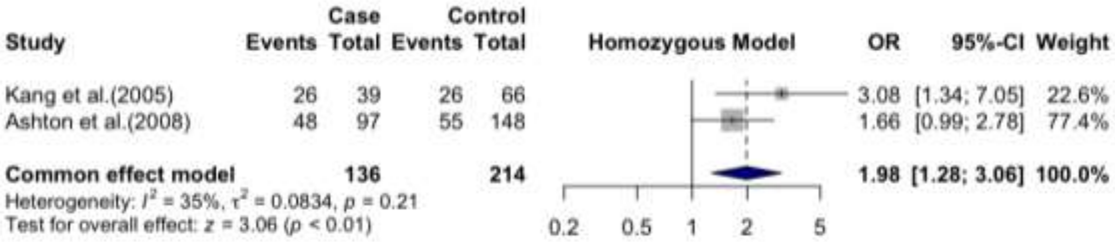

g) rs1052133 (OGG1)

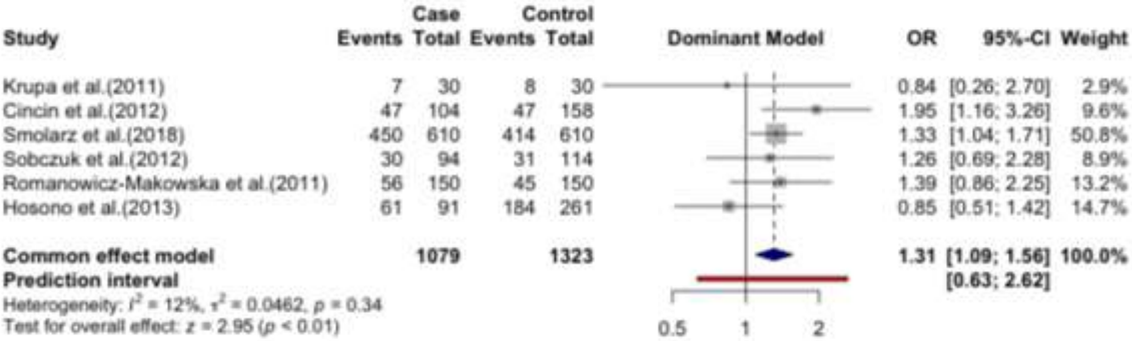

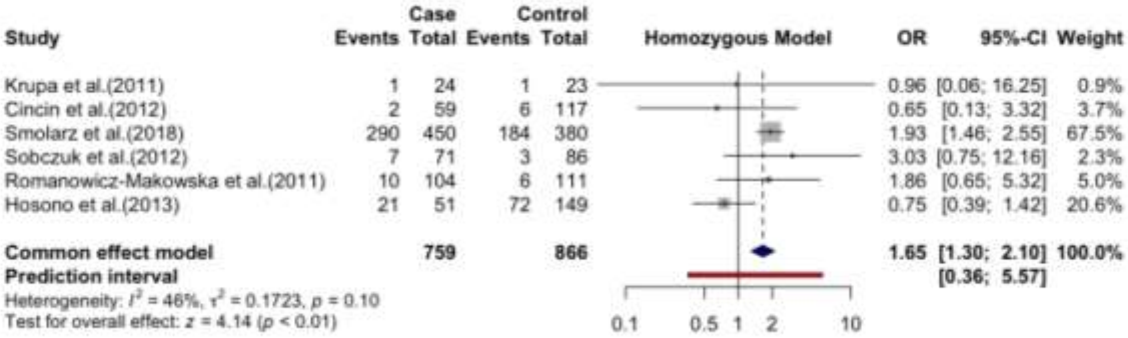

h) rs11224561 (PGR)

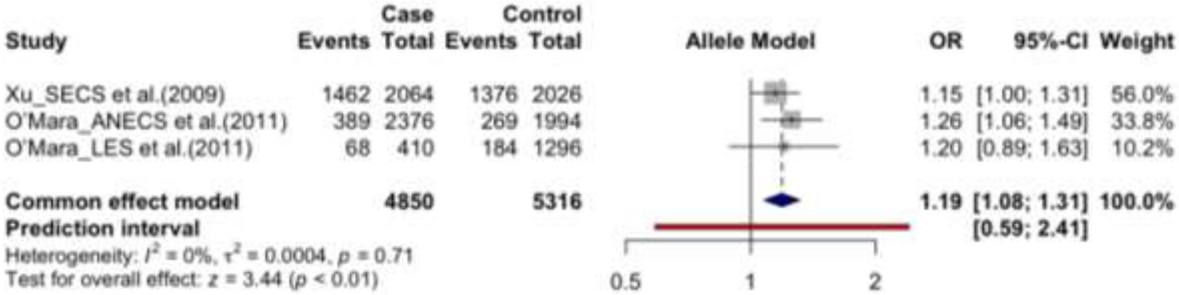

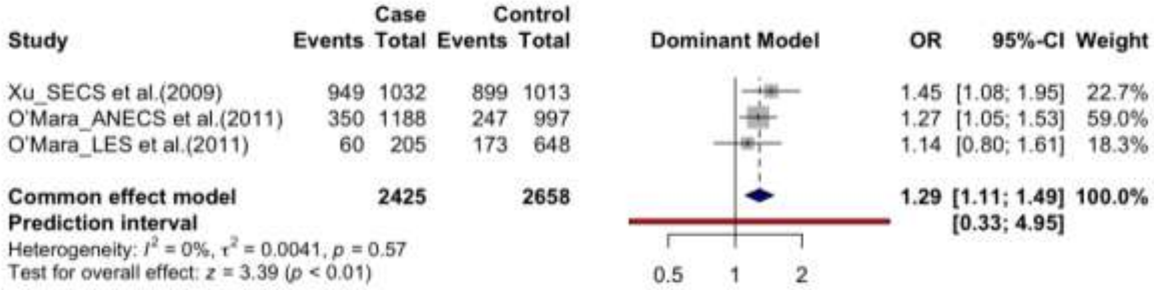

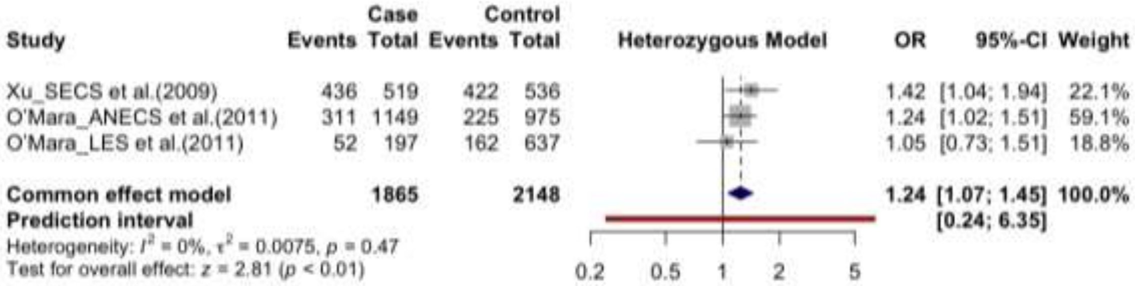

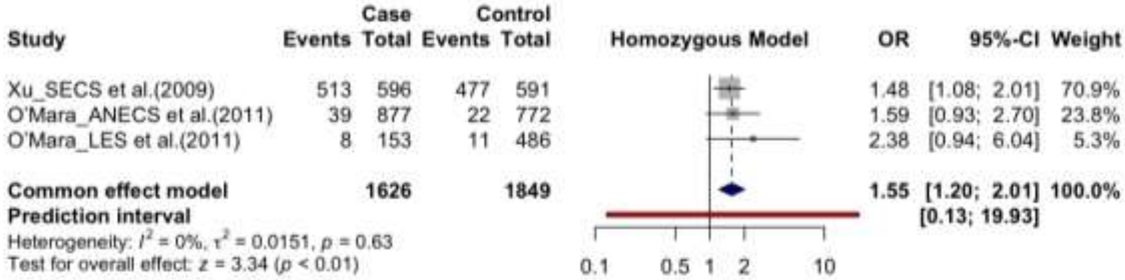

i) rs1042028 (SULT1A1)

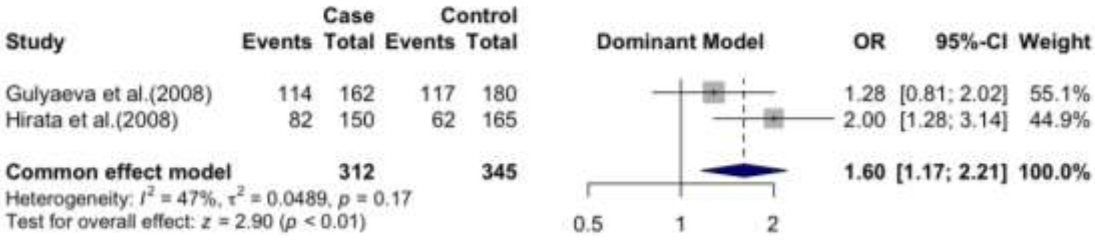

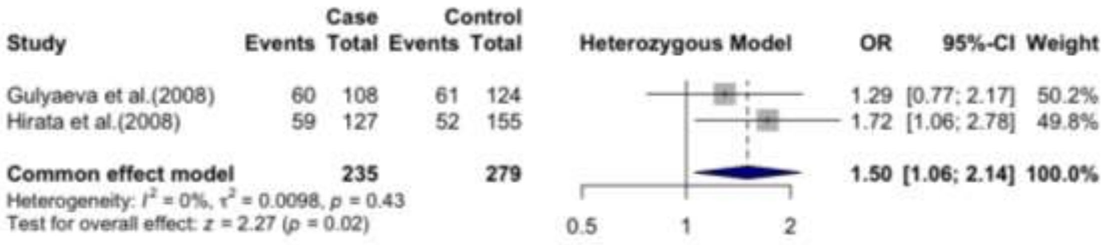

j) rs1800734 (MLH1)

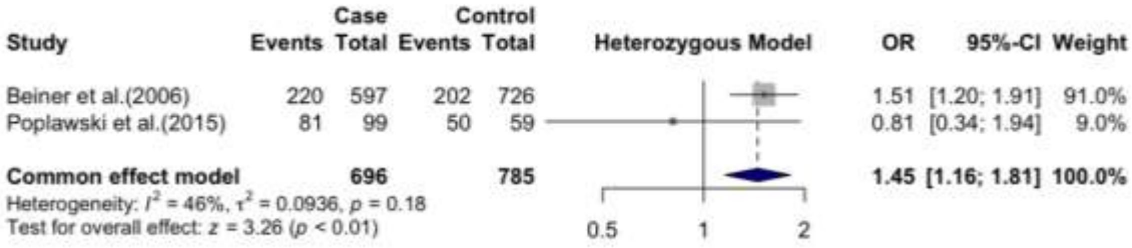

k) rs1799793 (ERCC2)

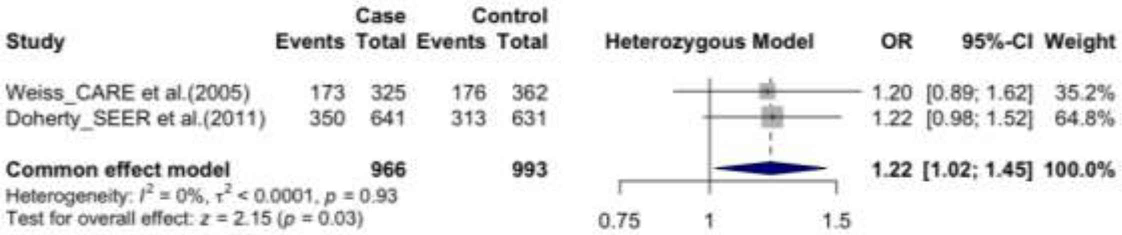

Supplement: Supplementary file 1 [file genes-14-00741-s001.zip › Supplementary File1.pdf]
